# Visible-Light Photoredox Catalysis in Water

**DOI:** 10.1021/acs.joc.2c00805

**Published:** 2022-06-14

**Authors:** Camilla Russo, Francesca Brunelli, Gian Cesare Tron, Mariateresa Giustiniano

**Affiliations:** † Department of Pharmacy, University of Naples Federico II, via D. Montesano 49, 80131 Napoli, Italy; ‡ Department of Drug Science, University of Piemonte Orientale, Largo Donegani 2, 28100 Novara, Italy

## Abstract

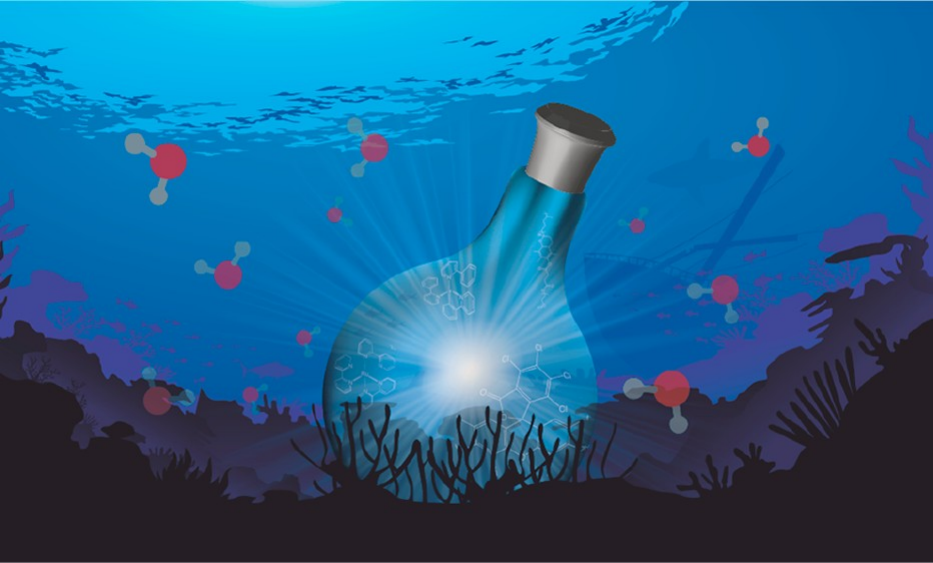

The use of water in organic synthesis draws attention to its green chemistry features and its unique ability to unveil unconventional reactivities. Herein, literature about the use of water as a reaction medium under visible-light photocatalytic conditions is summarized in order to highlight challenges and opportunities. Accordingly, this Synopsis has been divided into four different sections focused on (1) the unconventional role of water in photocatalytic reactions, (2) *in*-/*on-*water reactions, (3) water-soluble photocatalysts, and (4) photomicellar catalytic systems.

Wohler’s synthesis of urea, developed in 1828 and performed by heating an aqueous solution of ammonium cyanate, is commonly considered the starting point for synthetic organic chemistry. Similarly, many name reactions developed in the 19th century, e.g., the Curtius rearrangement, the Pictet–Spengler reaction, and the Sandmeyer reaction, to cite a few, were first developed in aqueous media.^1^ At the beginning of the 20th century, the advent of organometallic chemistry, with the Grignard reagents, made the switch to the era of organic solvents, which started to be readily available thanks to the rapidly expanding petroleum industry. The organomagnesium halides, indeed, were decomposed by water “with violence”, thus requiring rigorously anhydrous conditions. Nevertheless, Nature chooses water as a solvent to perform many elegant biochemical transformations that rely on the exploitation of hydrophobic energies to promote substrate binding into enzyme pockets and drugs into receptors. In the 1980s, Breslow, fascinated by the possibility of exploiting such a *hydrophobic effect* to accelerate cycloaddition reaction rates, reported his seminal results about the Diels–Alder reaction.^2^ His pioneering studies marked the dawn of a renewed interest in the use of water as a reaction medium. Water, indeed, meets many green chemistry principles: it is inflammable, incombustible, nontoxic, cheap, readily available, polar, and easy to separate from nonpolar immiscible organic solvents. In addition to these green features, water is unique for its exclusive ability to trigger new reactivities relying on unconventional reaction mechanisms. While literature about water as a reaction medium for organic synthesis has been collected in both books and authoritative reviews,^1,3^ the potential and limitations of its use in visible-light photocatalytic transformations have received little attention so far. Visible-light photocatalysis represents one of the most exciting and flourishing fields in organic synthesis by virtue of the possibility of generating open-shell species under very mild reaction conditions and exploiting them in many transformations not always achievable via ground-state reactivities. Importantly, the latter also involve the late-stage editing of complex molecular architectures such as drugs, thus leading visible-light photocatalysis to cross the limits of academia and become a powerful tool for many pharmaceutical companies to either generate compounds in a more efficient manner or to gain access to completely new and patentable chemical entities.^4^ Additionally, photoredox catalysis can be considered a green chemistry tool: light is a free renewable energy source, the photocatalysts are used in low amounts, no high temperatures, pressures, or harsh conditions are required so that less hazardous operating conditions, and minor energy consumption are made available.^5^ At the same time, the high chemoselectivities and functional group orthogonality facilitate purification steps and waste disposal by minimizing the formation of unwanted side products. Accordingly, merging the green innate properties of photoredox catalysis with the use of water as a reaction medium would boost the development of sustainable organic processes available for both drug manufacturing and materials chemistry. In addition, the exclusive ability of water to unravel new chemical behaviors and to drive unconventional reaction pathways breaks new ground for future advancements in the field. The aim of this Synopsis is to shed light on the opportunities and challenges offered by visible-light photoredox catalysis in water. Recently, Kobayashi drew attention to the urgent need of a conceptual classification of chemical processes performed by using water as the sole reaction medium and proposed to adopt *in water reactions* as a general term when water is used as a reaction medium.^6^ According to Lipshutz, depending on the conditions, water could promote *on water* reactions (i.e., when no solvation of the reagents occurs), *with water* reactions (i.e., water present within the medium), and *in water* reactions (where the use of additives enables the solubilization of starting materials and catalysts).^7^ The literature in the field has been divided herein in four different sections focused on (1) the unconventional role of water in photocatalytic reactions, (2) *in*-/*on-*water reactions, (3) water-soluble photocatalysts, and (4) photomicellar catalytic systems.

## 1 Water Is Special: Unconventional Roles in Visible-Light Photocatalytic Reactions

### 1.1 Water and Electron Donors as Reductive Equivalents

**Scheme 1:**
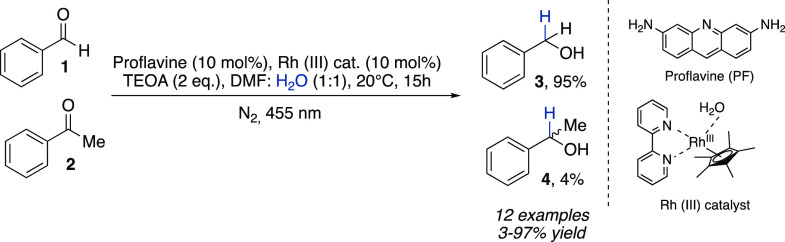
Chemoselective Photoreduction of Aldehydes in the Presence of Ketones

Light-driven reduction of organic substrates by harnessing water and an electron donor (such as a tertiary aliphatic amine) as reductive equivalents and an organo-metallic or a coordination complex as a hydride-transfer agent could be classified as cooperative photoredox catalysis.^8^ A seminal report is represented by the chemoselective photoreduction of aldehydes such as benzaldehyde **1** (Scheme 1) in the presence of ketones (e.g., **2**) reported by König in 2015.^9^ The authors used triethanolamine (TEOA) as a sacrificial electron donor, proflavine (PF) as the photocatalyst, and [Cp*Rh(III)(bpy)Cl]Cl (Rhcat) as the mediator, while the effective reducing agent was found to be [Cp*Rh(III)(bpy)H]Cl (Rh(III)-H) (Scheme 1). This study highlights the role of water as the hydride source when combined with an electron donor under visible-light irradiation, although a DMF/H_2_O 1:1 is required to achieve optimum yields.

**Scheme 2:**
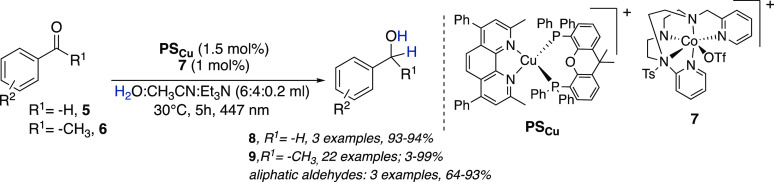
Light-Driven Reduction of Aldehydes and Aromatic Ketones

Recently, Lloret-Fillol and co-workers reported a light-driven reduction of aldehydes **5** (Scheme 2) and aromatic ketones **6** by using a dual metal catalyst formed by an aminopyridine cobalt complex **7**, forming a [Co–H] intermediate, and [Cu(bathocuproine)(Xantphos)](PF_6_) **PS**_**Cu**_ as photoredox catalyst (Scheme 2).^10^ This methodology, relying on earth-abundant elements, uses water and an electron donor (Et_3_N or ^*i*^Pr_2_EtN) as the hydride source and is performed in aqueous mixtures (80–60% water) with exquisite selectivity toward aryl ketones **6** in the presence of terminal olefins, both aliphatic ketones and aldehydes, and alkynes.

### 1.2 Enhancing the Reductive Power of Ruthenium Photocatalysts

**Scheme 3:**
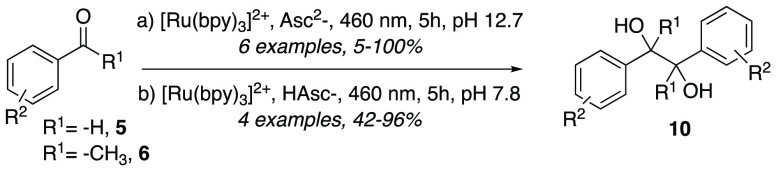
Photocatalytic Pinacol Coupling Alternatively, [Ru(dmb)_3_]^2+^ was used as PC; dmb: tris(4,4′-dimethyl-2,2′-bipyridine)ruthenium(II).

Tris(2,2′-bipyridine)ruthenium(II) Ru(bpy)_3_^2+^ is among the most popular photocatalysts thanks to its long-lived metal-to-ligand charge-transfer (MLCT) excited state and the feasibility of both oxidative and reductive catalytic cycles. However, based on its standard potential (Ru^III^/Ru^II^ −1.29 V vs SCE),^11^ it is unable to promote more demanding transformations, which thus require more expensive and higher energy iridium complexes. Goez and Naumann, while investigating the feasibility of a pinacol coupling approach under sustainable photocatalytic conditions, found that the one-electron reduced forms (OER) of Ru(bpy)_3_^2+^ exhibited a reductive power greater by 0.2 eV in water than in acetonitrile.^12^ This chemical behavior was also confirmed by laser flash photolysis studies. The catalytic system exploited ascorbate as a sacrificial donor and, in case of hydrophobic starting materials, SDS micelles or cyclodextrins to overcome solubility problems (Scheme 3).

### 1.3 Role of Water in Reductive Proton Transfer from Carbonyls to Alcohols

**Scheme 4:**
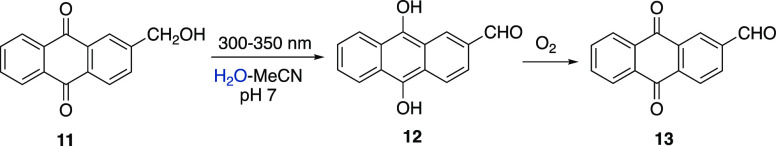
Intramolecular Reductive Proton Transfer from Carbonyls to Alcohols

Wan et al. showed that water could also have a key role to promote a formal intramolecular photoredox reaction of 2-(hydroxymethyl)anthraquinone (**11**, Scheme 4) and other analogues.^13^ When 2-(hydroxymethyl)anthraquinone **11** was irradiated in water with UV light sources a formal intramolecular redox product **12** formed, which readily oxidized to anthraquinone **13** under air or oxygen (Scheme 4).

### 1.4 Water-Promoted Rate Acceleration

**Scheme 5:**
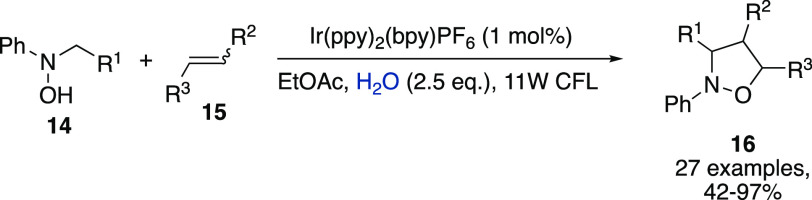
Iridium-Promoted Synthesis of Isoxazolidines

It is not uncommon to find visible-light photoredox catalytic synthetic protocols where optimum reaction conditions require the addition of amounts of water—ranging from a few equivalents to precise volume ratios—to either miscible (e.g., CH_3_CN, DMF, DMSO) or immiscible (e.g., DCM, EtOAc) organic solvents. Representative reports where the presence of water in the reaction medium proved to accelerate the reaction rate involve the visible-light photocatalytic iridium-promoted synthesis of isoxazolidines **16** by Rueping et al. (Scheme 5).^14^ In the search for experimental evidence supporting the mechanistic basis of such rate acceleration, the authors hypothesized that a photoredox promoted water splitting could have provided active species such as HO^•^, HO^–^, and H_2_O_2_. However, this hypothesis was ruled out while the cycloaddition reaction could benefit from a catalytic role of the species such as, e.g., H_3_O^+^ and H_2_O, present in the reaction mixture.

**Scheme 6:**
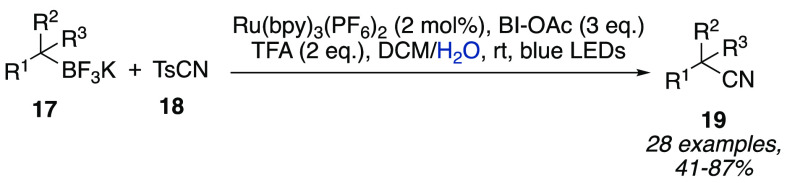
Deboronative Cyanation

Unfortunately, most of the synthetic protocols involving water either as additive or cosolvent did not investigate the mechanistic rational underpinning the beneficial effect of water. It is worth noting that many useful radical precursors such as alkyl and aryl bromides, diazonium salts, carboxylic acids and esters, and trifluoroborate salts,^15^ to cite a few, are stable in neutral aqueous conditions. As a matter of example, the latter have been involved in a deboronative cyanation reaction promoted by a ruthenium-based photocatalyst, a hypervalent iodine oxidant, a cyanide source, and TFA as an additive (Scheme 6). The different nature of the starting materials required a biphasic 1:1 DCM/H_2_O solvent system, whereas 2:1 mixtures of organic solvents such as acetone/DCM and HFIP/DCM led to decreased yields.^16^

### 1.5 LUMO-Lowering Effect

**Scheme 7:**
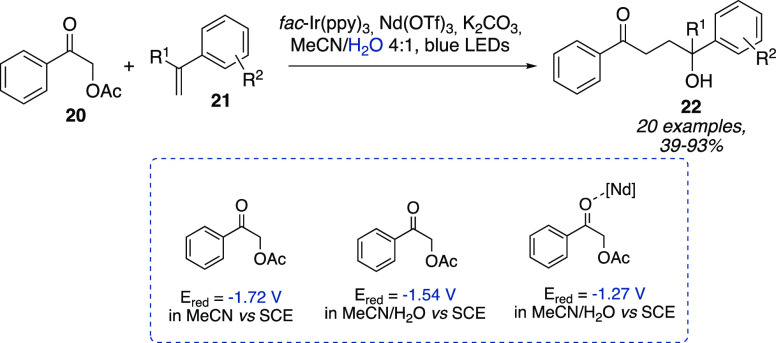
Photocatalytic Cross-Coupling of α-Carbonyl Acetates with Styrenes

Among the exclusive features of water, Zeitler et al. reported a LUMO lowering effect observed for α-carbonyl acetates **20** as radical precursors in the photocatalytic cross-coupling with styrenes **21** (Scheme 7).^17^ In more detail, the authors employed *fac*-Ir(ppy)_3_ as a photocatalyst to promote the formation of an alkyl radical from α-acetylated acetophenone derivatives **20** (Scheme 7). The latter represented challenging substrates due to their redox potential (*E*_red_= −1.72 V in MeCN vs SCE). Interestingly, the presence of water proved to induce a LUMO lowering effect by increasing the *E*_red_ to −1.54 V vs SCE, effect that was still enhanced with the use of a water-compatible Lewis acid such as Nd(OTf)_3_ (*E*_red_ = −1.27 V vs SCE). In the absence of the Lewis acid, the 1,4-difunctionalized product was not detected.

### 1.6 Water Influencing Chemoselectivity

**Scheme 8:**
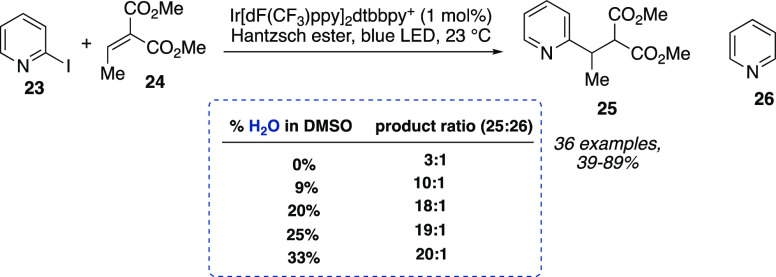
Radical Conjugate Addition of Nitrogen Heterocycles to Electron-Poor Alkenes

Besides acting as a solubilizing agent for polar substrates, water could also be harnessed for its nonsolvent properties toward lipophilic hydrophobic additives and/or reagents. A remarkable example of such ability was reported by Jui and co-workers for the radical conjugate addition of nitrogen heterocycles **23** to electron-poor alkenes **24** (Scheme 8). By performing the reaction in DMSO with percentages of water spanning from 0 to 33% the authors demonstrated how the limiting reactant solubility, i.e., the Hantzsch ester (HE), improved selective formation of the Michael addition products **25** over the formation of the reduced nitrogen heterocycles **26** (Scheme 8).^18^

**Scheme 9:**
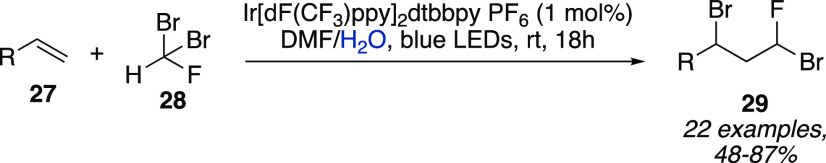
Chemoselective Synthesis of Hydro- and Dibromofluoromethylated Adducts

More recently, Qing described how the switch from THF to a DMF/H_2_O mixture enabled the chemoselective synthesis of hydro- and dibromofluoromethylated adducts **29** (Scheme 9), respectively, starting from alkenes **27** and dibromofluoromethane **28**.^19^ By performing the reaction in THF-*d*_8_ deuterated product was formed, thus confirming the role of THF as the hydrogen source. On the other hand, while the use of the sole DMF as the solvent still afforded a mixture of both hydro- and dibromofluoromethylated products, the use of a 1:4 DMF/H_2_O solvent system completely suppressed the formation of the hydrobromofluoromethylated adduct.

## 2 In-/On-Water Reactions

**Scheme 10:**
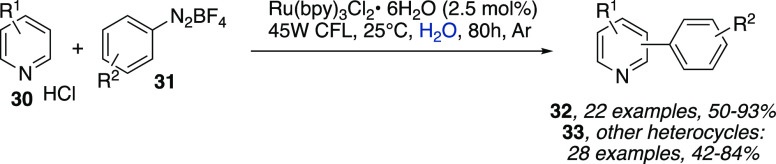
Direct Arylation of *N*-Heteroarenes with Aryldiazonium Tetrafluoroborate Salts

**Scheme 11:**
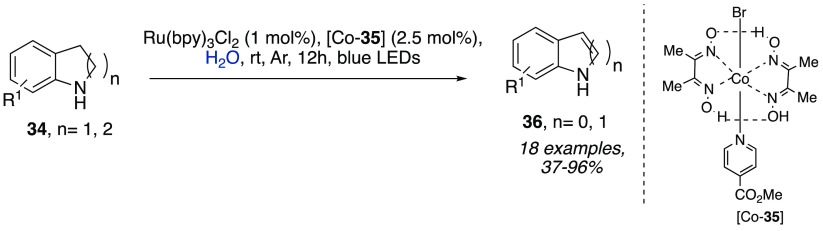
Catalytic Dehydrogenation of Cyclic Amines

**Scheme 12:**
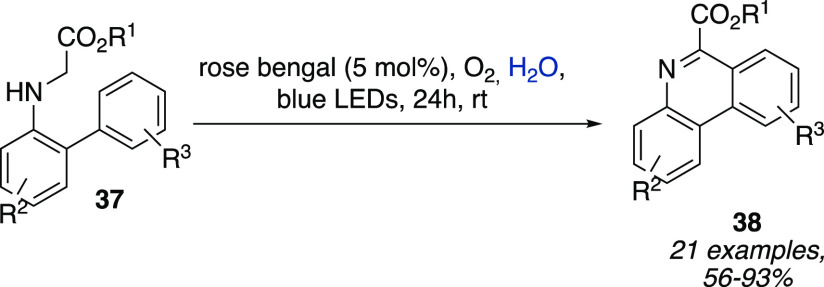
Metal-Free Oxidative Radical Cyclization of *N*-Biarylglycin Esters

A number of visible-light photoredox catalytic transformations have been reported *in water* albeit involving slightly or not soluble reagents/additives/photocatalysts. Accordingly, it is quite difficult to classify them as *real in water* or *on water reactions* as proposed by Lipshutz.^3^ For instance, such photochemical methodologies included the direct arylation of *N*-heteroarenes **30** with aryldiazonium tetrafluoroborate salts **31** as radical precursors and [Ru(bpy)_3_]^2+^ as the photoredox catalyst, both poorly soluble in water (Scheme 10),^20^ the catalytic dehydrogenation of cyclic amines **34** promoted by a ruthenium-based photocatalyst and a cobalt-based cocatalyst [Co-**35**] (Scheme 11),^21^ and the metal-free oxidative radical cyclization of *N*-biarylglycin esters **37** leading to phenanthridine-6-carboxylate derivatives **38** (Scheme 12).^22^

**Scheme 13:**
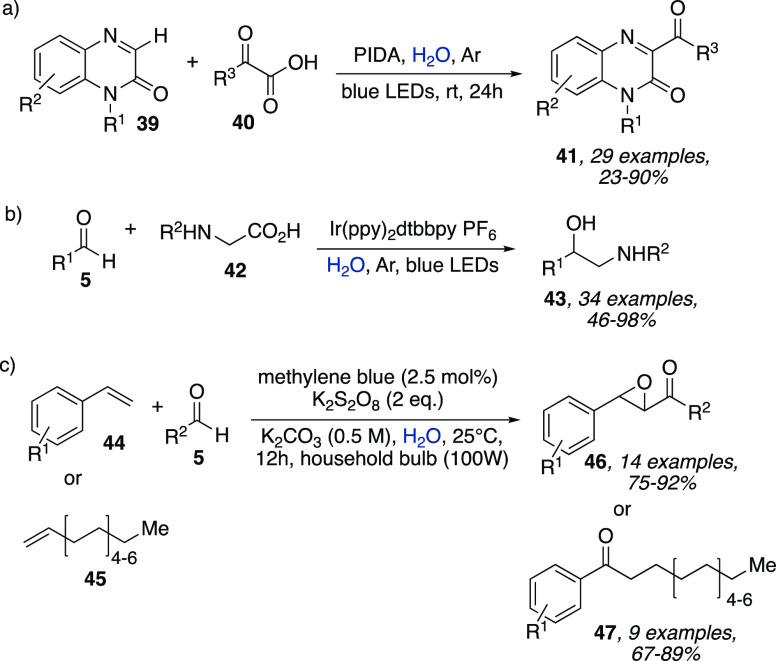
(a) C3–H Acylation of Quinoxaline-2(1*H*)-ones **41**. (b) Synthesis of 1,2-Amino alcohols **43**. (c) Epoxyacylation and Hydroacylation of Olefins **44** and **45**

Other interesting visible-light-assisted methodologies were represented by the C3-H acylation of quinoxaline-2(1*H*)-ones **39**,^23^ the synthesis of 1,2-amino alcohols **43** by decarboxylative coupling of amino acids **42** derived α-amino radicals to carbonyl compounds **5**,^24^ and the epoxyacylation and hydroacylation of olefins **44** and **45** using methylene blue and persulfate as the oxidant (Scheme 13).^25^ In all these cases, the use of water as solvent demonstrated to be superior to common organic solvents such as MeCN, DMSO, MeOH, and DMF. Unfortunately, the reasons why water led to an increase in the reaction yields were not investigated in detail.

More recently, the role of water as reaction medium for photodimerizations was highlighted by Ramamurthy et al. in light-promoted cycloaddition reactions of sparingly water-soluble compounds such as coumarin, indene, cinnamic acid, and acenaphthylene. The observed increased reactivity with respect to organic solvents was ascribed to hydrophobic association due to poor solubility of the substrates, which, however, required irradiation of large volumes to collect reasonable amounts of the products.^26^

## 3 Water-Soluble Photocatalysts

**Figure 1:**
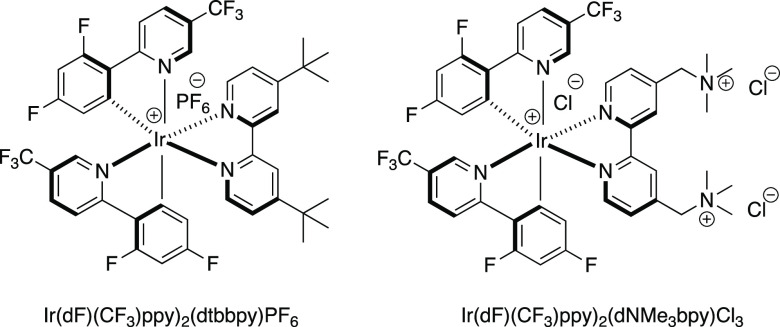
[Ir(dF(CF_3_)ppy)_2_(dtbbpy)]PF_6_ and its water-soluble analogue [Ir(dF(CF_3_)ppy)_2_(dNMe_3_bpy)]Cl_3_.

Most of iridium– and ruthenium–polypyridyl complexes used as visible-light photoredox catalysts show very poor water solubility (from <1 to 1000 ppm) depending on both their substitution pattern and the anionic partner.^27^ Curiously, even in organic solvents, they are often used at loadings exceeding their maximum solubility, probably limiting the reaction efficiencies as recently investigated by Jespersen et al.^27^ On the other hand, the development of visible-light photocatalysts enabling homogeneous conditions in water represents an attractive field of investigation including, for example, recent reports by Roelfes et al.^28^ and Conrad et al.^29^ developing water-soluble iridium photoredox catalysts by changing the dative ligands (e.g., *tert*-butyl) in [Ir(dF(CF_3_)ppy)_2_(dtbbpy)]PF_6_ with either quaternary ammonium functional groups or carboxylic acid residues, respectively. The Roelfes catalyst, [Ir(dF(CF_3_)ppy)_2_(dNMe_3_bpy)]Cl_3_ (Figure 1), was harnessed to modify dehydroalanine (Dha) residues in peptides and proteins, in water and under physiologically relevant conditions, with short reaction times and with low reagent and catalyst loadings.^28^

**Scheme 14:**
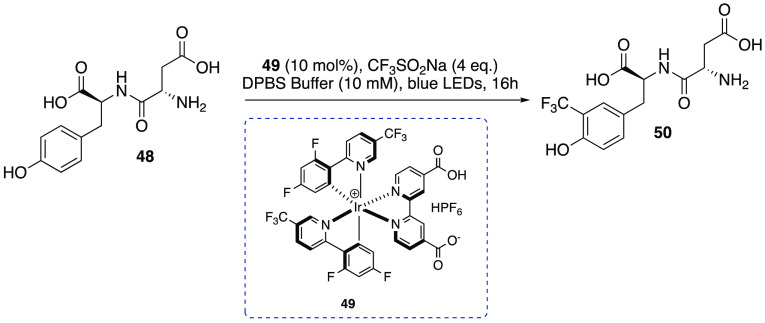
Trifluoromethylation of Unprotected Dipeptide **48** in Phosphate-Buffered Saline Solvent

Ligand manipulation of the same iridium complex by introducing carboxylic acid moieties on the bipyridyl ligand led to catalyst **49** with improved water solubility but with similar oxidation potential thanks to the retention of fluoro and trifluoromethyl functional groups (*E*_1/2red_[*Ir^III^/Ir^II^] = +0.76 V vs SCE for the hemisalt [Ir(dF(CF_3_)ppy)_2_((CO_2_H)(CO_2_)bpy)]_2_·HPF_6_) (Scheme 14). Such a photocatalyst was applied to the trifluoromethylation of acyl tyrosine amide and an unprotected dipeptide Asp-Tyr (**48**, Scheme 14) in phosphate-buffered saline solvent (DPBS) using Langlois reagent as the CF_3_ radical source under blue LED light irradiation.^29^

**Scheme 15:**
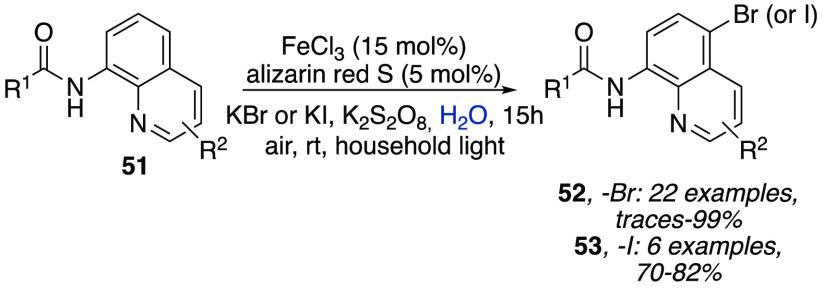
Bromination and Iodination of 8-Aminoquinoline Amides in Water

Despite their excellent photoredox properties, the use of precious metal-based catalysts represents a huge limitation to the green potential of chemical transformations induced by renewable energy sources such as visible light. In the search for more sustainable protocols, Wu et al. achieved bromination and iodination of 8-aminoquinoline amides in water by merging FeCl_3_ with a water-soluble organic photocatalyst such alizarin red S.^30^ The reaction also required K_2_S_2_O_8_ as the oxidant and KBr as an additive and proceeded at room temperature under air and household light irradiation to afford halogenated quinolines **52** and **53** (Scheme 15) in good to excellent yields regardless of the presence of electron-donating or electron-withdrawing groups on the benzamide ring.

**Scheme 16:**
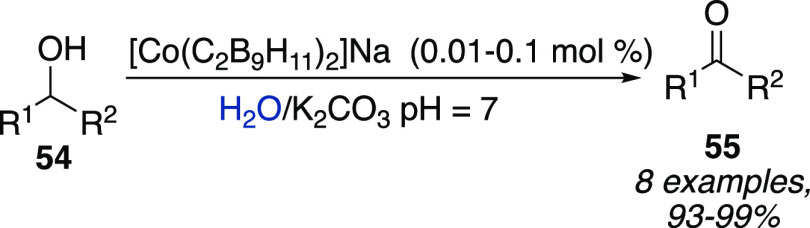
Oxidation of Aromatic and Aliphatic Alcohols in Water

Romero, Teixidor et al. focused on metallacarboranes systems, describing that very low loadings of [Co(C_2_B_9_H_11_)_2_]^−^ (0.1 to 0.01 mol %) could promote the oxidation of aromatic and aliphatic alcohols **54** in water with up to quantitative conversion thanks to their high solubility (Scheme 16). Interestingly, the addition of [NMe_4_]Cl enabled an easy recovery of the photocatalyst by precipitation.^31^

**Scheme 17:**
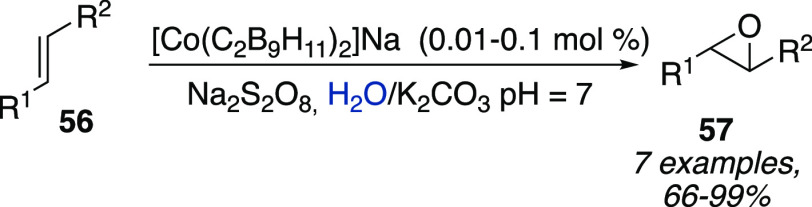
Oxidation of Alkenes **56** to Epoxides **57** in water

The same photocatalytic system was also applied to the oxidation of alkenes **56** to epoxides **57** in water, whereas the widely used photosensitizer tris(2,2′-bipyridine)ruthenium(II) ([Ru(bpy)_3_]^2+^) showed very low efficiency. Actually, catalyst loading of 0.01 mol % enabled conversions between 65 and 97% in short reaction times (15 min) and with excellent chemoselectivity toward the formation of epoxide derivatives **57** (with respect to diols side products) (Scheme 17).^32^

**Scheme 18:**
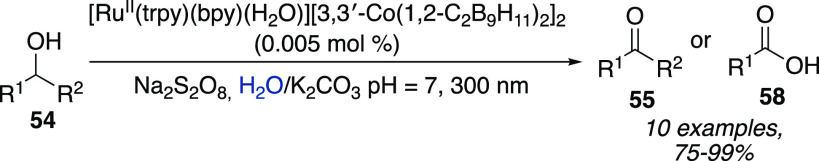
Photooxidation of Alcohols to Aldehydes and Carboxylic Acids

Such a metallacarborane was also covalently linked on magnetic nanoparticles (MNPs) coated with a silica layer to provide a heterogeneous catalytic system endowed with easy magnetic separation and recyclability, which proved to efficiently photooxidize alcohols using a loading of 0.1 and 0.01 mol %.^33^ More recently, the same authors developed a cooperative ruthenium cobaltabis(dicarbollide) photoredox catalytic system [Ru^II^(trpy)(bpy)(H_2_O)][3,3′-Co(1,2-C_2_B_9_H_11_)_2_]_2_ (trpy = terpyridine and bpy = bipyridine) where the photoredox metallacarborane and the ruthenium oxidation catalyst were linked by noncovalent interactions persisting even after water dissolution (Scheme 18).^34^ The photooxidation of alcohols **54** to aldehydes **55** and carboxylic acids **58** was accomplished via a proton-coupled electron-transfer process under UV light irradiation and with a 0.005 mol % of catalyst loading (Scheme 18).

**Scheme 19:**
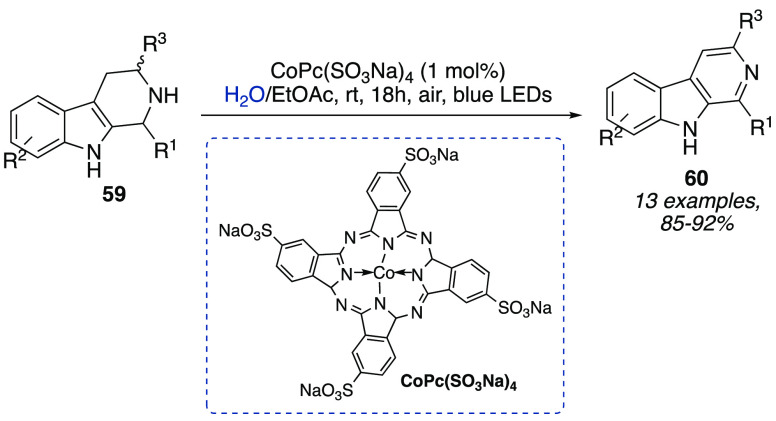
Oxidative Dehydrogenation of *N*-Heterocycles in a Biphasic System

A cobalt-based phthalocyanine photoredox catalyst [CpPc(SO_3_Na)_4_] enabling oxidative dehydrogenation of *N*-heterocycles in a biphasic system was reported by Baskar et al. Namely, tetrahydro-β-carbolines **59**, indolines, and tetrahydro-(iso)quinolines were converted into biologically active indoles, (iso)-quinolines, and β-carbolines **60** (Scheme 19).^35^ The use of a biphasic reaction medium afforded an easy separation and an efficient reusability of the catalyst (up to 5 times with almost comparable reactivity). The optimum reaction conditions were also amenable to gram scale.

On the other hand, the selective oxidation of aromatic alcohols in water under visible-light organocatalytic conditions was pioneered by Wang et al. in 2013 by using mesoporous graphitic carbon nitride (mpg-CN), a metal-free polymeric photocatalyst able to activate molecular oxygen for selective oxygenation or oxidative dehydrogenation of organic substrates.^36^ Such a catalytic system was endowed with high tunability of a wide range of parameters and both higher conversion and higher selectivity to the aldehyde when compared with other widely investigated visible-range inorganic photocatalysts.

## 4 Photomicellar Catalytic Systems

**Scheme 20:**
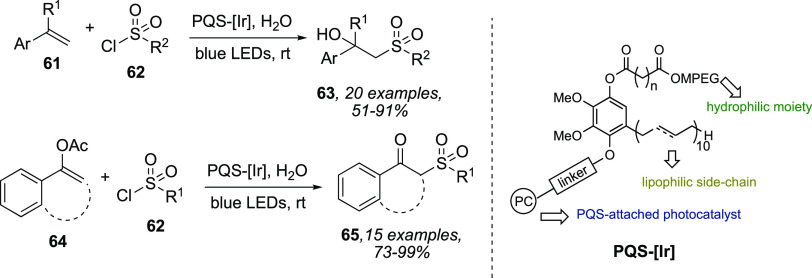
Difunctionalization of Alkenes **61** and Sulfonylation of Enol Acetates **64** Promoted by PQS-[Ir]

If the development of water-soluble photocatalysts would promote new chemical transformations of polar substrates such as amino acids, peptides, and proteins, most of the synthons used in drugs’ manufacturing require organic solvents for their dissolution, thus limiting the green chemistry features of these chemical approaches. A solution to this problem could arise from the application of micellar solutions as the reaction medium to photoredox catalytic conditions. Micellar catalysis, pioneered by Lipshutz, has been receiving increasing attention thanks to promising results reported in literature.^37^ The same Lipshutz in 2018 reported the design and the synthesis of a PQS-covalently (PQS = polyethyleneglycol ubiquinol succinate, reduced form of dietary supplement CoQ_10_) linked iridium photocatalyst **PQS-[Ir]** undergoing self-aggregation in water into nanomicelles.^38^ The efficiency of the amphoteric catalyst was demonstrated with different representative reactions involving difunctionalization of alkenes **61** and sulfonylation of enol acetates **64** (Scheme 20). Notably, the aqueous reaction mixture could be easily recycled *in-flask* up to 10 times without significant losses in the reaction yields.

**Scheme 21:**
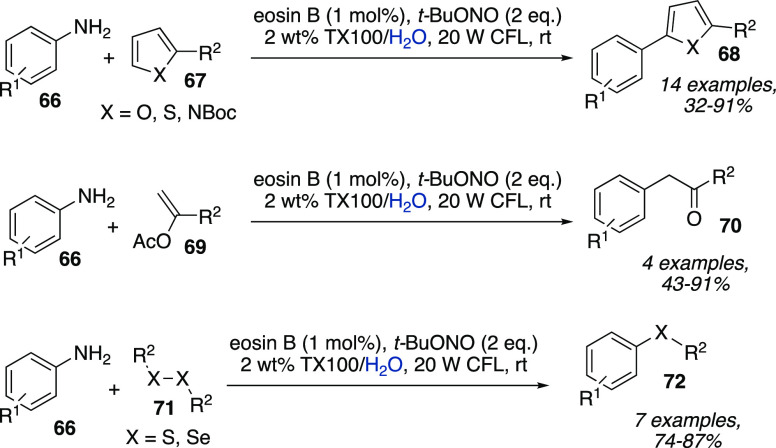
Arylation of Heteroarenes and Enol Acetates and Synthesis of Sulfides and Selenides

Notwithstanding the obvious and exclusive advantages offered by the micellar photocatalytic system developed by Lipshutz, the need for the availability of photocatalysts endowed with a wide range of redox potentials to promote chemical transformations starting from a plethora of radical precursors, and on the other hand, the need for a preliminary design and synthesis of photoredox catalysts covalently linked to surfactants, together with their commercial unavailability, could represent a limit to a popular application. Probably, these are the reasons why most of the recent literature reports focus on the optimized use of well-known both metal-based and organic photoredox catalysts in common aqueous micellar solutions such as TPGS-750M, Triton-X, SDS, and CTAC, to cite a few. For instance, Cai et al. accomplished arylation reactions with diazonium ions generated in situ by using eosin B in a 2 wt % Triton-X solution in water without any cosolvents or additives at room temperature (Scheme 21).^39^*tert*-Butyl nitrite was used as the nitrosating agent, and the optimum conditions afforded arylation of a wide range of anilines **66**, with electron-poor substrates giving higher yields with respect to electron-rich ones. Suitable heteroarenes **67** included furan, thiophene, and Boc-pyrrole. The developed photomicellar catalytic system also proved to efficiently promote α-arylation of enol acetates **69** and the synthesis of sulfides and selenides **72** (Scheme 21).

**Scheme 22:**
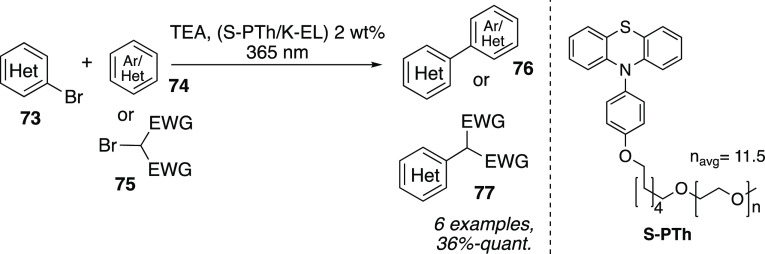
Direct Arylations of Aryl Bromides in Water

Similar products can be obtained via direct arylations of aryl bromides **73** in water in a microfluidic reactor under irradiation with UV light as recently reported by Mattiello, Beverina, et al. (Scheme 22).^40^ The authors investigated in detail the catalytic efficiency of an association colloid formed in water by castor oil derivative Kolliphor EL (K-EL) and a specifically devised photoredox active surfactant (**S-PTh**). It was shown that the use of surfactants enabled a good selectivity with respect to the competing dehalogenation process.

**Scheme 23:**
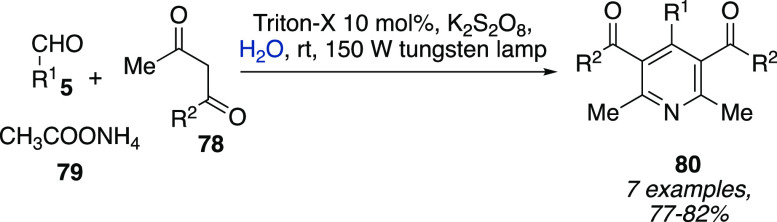
One-Pot Synthesis of Pyridines

Aromatization of 1,4-dihydropyridines promoted by visible-light irradiation, K_2_S_2_O_8_ as oxidant, in an aqueous solution of Triton-X was proposed in 2013 by Das et al. (Scheme 23).^41^ Performing the reaction one-pot starting from an aldehyde **5**, ethyl acetoacetate **78**, and ammonium acetate **79** in the presence of visible-light irradiation was proposed as a greener method to synthesize pyridine derivatives **80** in very good yields (Scheme 23).

**Scheme 24:**
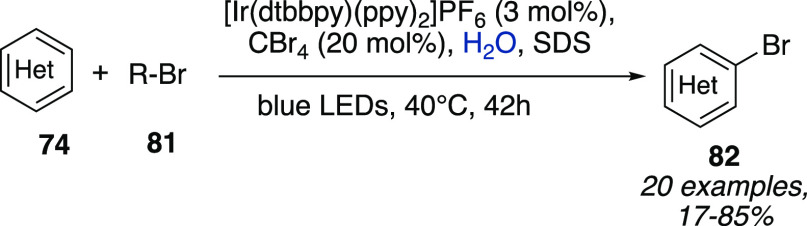
Photomicellar Catalytic Minisci C–H Functionalization of Heteroarenes

A Minisci C–H functionalization of heteroarenes **74** with nonactivated alkyl bromides **81** under photomicellar catalytic conditions was reported by Giedyk et al. (Scheme 24).^42^ The merging of bromide anion cocatalysis with photoredox catalysis in SDS micellar aqueous solution allowed to avoid stoichiometric radical promoters, oxidants, and acids as additives. It was shown how the spatial preaggregation of reacting species was key to promote the desired transformation since a very different outcome was observed depending on the use of neutral, anionic, or cationic surfactants. Interestingly, the same reaction performed in MeCN instead of SDS led to no product, further highlighting the critical importance of microstructuring of the components in the reaction medium.

**Scheme 25:**
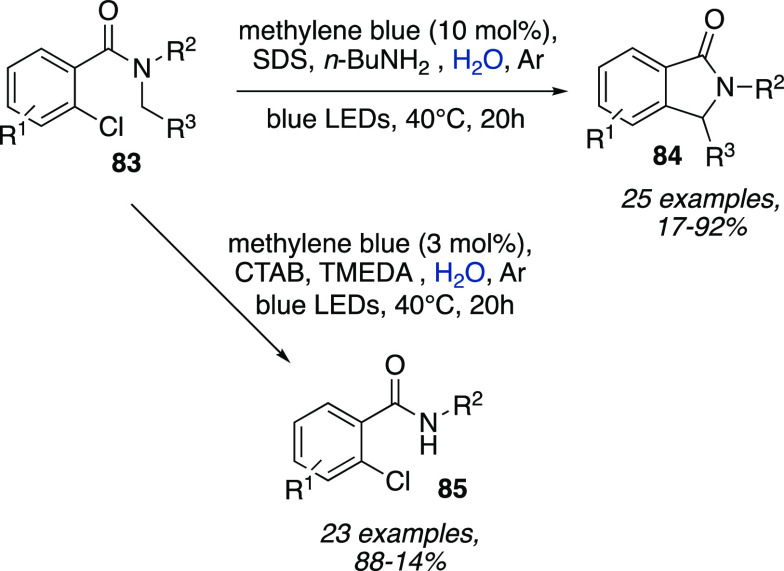
Chemodivergent Intramolecular C–H Arylation and *N*-Dealkylation of *o*-Chlorobenzamides **83**

Interestingly, the same authors showed how the use of an anionic surfactant rather than a cationic one could be the key for chemoselectivity. Irradiation of *o*-chlorobenzamides **83** with blue LEDs, in the presence of methylene blue as the photocatalyst and an amine as a sacrificial electron donor (TMEDA or *n*-BuNH_2_), could lead to either intramolecular C–H arylation or *N*-dealkylation depending on the use of a CTAB or an SDS micellar solution as a reaction medium, respectively (Scheme 25).^43^

**Scheme 26:**
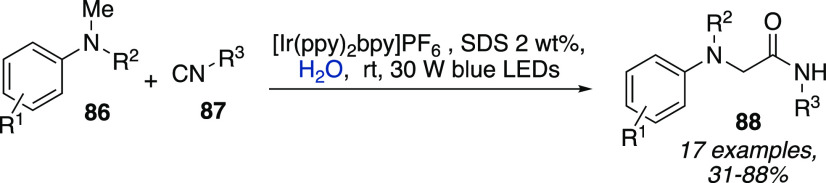
Photomicellar Catalytic Synthesis of Amides Starting from Isocyanides and Tertiary Aromatic Amines

Recently, we reported the synthesis of amide derivatives **88** starting from both aliphatic and aromatic isocyanides **87** and tertiary aromatic amines **86** using [Ir(ppy)_2_bpy]PF_6_ in a 2 wt % SDS water solution under irradiation with blue LEDs at room temperature (Scheme 26).^44^ In order to investigate the reaction environment at the atomic level (e.g., the localization of the photocatalyst with respect to the micelles), solution 1D- and 2D-NMR experiments were performed in the presence of paramagnetic probes. Such studies led to the identification of a *reverse polarity principle*, according to which a negatively charged surfactant such as SDS could provide the localization of a positively charged photocatalyst on the micelles’ surface, thus leading to an improved efficiency with respect to water alone.

SDS micelles were also shown to be the best performing surfactants in laser-induced Wurtz-type syntheses promoted by 2-aminoanthracene irradiation at 355 nm.^45^ These conditions readily produced hydrated electrons acting as “super-reductants”, rapidly converting chloro-organic substrates into carbon centered radicals, which underwent up to quantitative dimerization.

One more strategy to achieve visible light promoted reactions in water lies in the encapsulation of a photoredox catalyst into a nanosized molecular capsule, such as a V-shaped aromatic amphiphile.^46^ Alternatively, amphiphilic polymeric nanoparticles have been harnessed as small reactors to perform light-driven chemical reactions. Palmans et al., by incorporating a phenothiazine catalyst into the polymeric scaffold, were able to perform metal-free reduction and C–C cross-couplings upon exposure to UV light.^47^ A further investigation of two popular organic photocatalysts, i.e., 10-phenylphenothiazine and one based on an acridinium dye, covalently linked to six different amphiphilic polymers forming nanoparticles in aqueous solution, was lately described by the same authors.^48^

Colloidal platinum nanoparticles dispersed by polyvinylpyrrolidone were instead used to set up a photoredox system of water-soluble zinc porphyrin and an electron mediator able to promote the selective reduction of pyruvate to lactate (a raw material for biodegradable polymers).^49^

In conclusion, the potentialities and the challenges of harnessing water exclusive properties in light-driven reactions have been herein highlighted. Such unique features span from applications as reductive equivalents (in combination with an electron donor) to the ability of enhancing the reductive power of ruthenium photocatalysts, to reductive proton transfer, water-promoted acceleration, LUMO-lowering effect, and the possibility of influencing chemoselectivity. A number of *in*/*on*-*water* reactions have been reported and continue to receive increasing attention, pointing out the need for a more precise classification of these protocols. Finally, while water-soluble photocatalysts have a pivotal role to promote peptides and proteins bioconjugation under visible light irradiation, the use of non-water-soluble mediators in micellar aqueous media stands for a promising approach to overcome the need for organic solvents and to develop milder and more sustainable processes. Besides being an added value *per se*, the progresses of photochemistry in water could be even more important if considering the feasibility of photobiocatalytic cascades.^50^ Given the polyhedric nature of water in light promoted chemical reactions, key and exciting future advancements in the field are expected.
